# Analysis of three treatment methods for granulomatous lobular mastitis: a retrospective study in a single center

**DOI:** 10.3389/fonc.2025.1588836

**Published:** 2025-04-30

**Authors:** Boyu Shang, Tianyi Zhang, Chang Liu, Jiaxin Lu, Can Cui, Jing Feng, Yi Zhou

**Affiliations:** Department of Breast Surgery, The First Affiliated Hospital of Harbin Medical University, Harbin, China

**Keywords:** granulomatous lobular mastitis, clinical presentation, surgical treatment, triple anti-tuberculosis drug therapy, treatment methods

## Abstract

**Background:**

Granulomatous lobular mastitis (GLM) is a rare, benign inflammatory condition of the breast that is difficult to distinguish from breast cancer on the basis of clinical and imaging findings. GLM typically has a long disease course, and is difficult to treat and prone to recurrence. Furthermore, there is currently no standard treatment for GLM. The aim of this study was to compare the therapeutic effects and patient satisfaction scores of surgery, triple anti-tuberculosis drug therapy, and combination treatment against GLM.

**Methods:**

The medical records and follow-up data of GLM patients who underwent treatment at our center were retrospectively analyzed. Patients were divided into the treatment groups of surgery (group A), triple anti-tuberculosis drug therapy (group B), and combination therapy (group C). The demographic and clinical data, treatment outcomes, and patient satisfaction scores were compared among the three groups.

**Results:**

Median follow-up duration of the patients was 35.43 months (range, 13.27–67.90 months). There were 106, 109, and 88 patients in groups A, B, and C respectively. The cure rates were similar among the groups (P = 0.220), although treatment duration was longest for group B, followed by group C and group A (P < 0.001). Group B had the highest patient satisfaction scores (P < 0.001),whereas the recurrence rate was highest in Group A (P < 0.001). Furthermore, no severe adverse drug reactions or major postoperative complications were observed in any of the patients.

**Conclusions:**

Triple anti-tuberculosis drug therapy can effectively treat GLM patients with high patient satisfaction scores for potential application in clinical practice. The combination of surgery and triple anti-tuberculosis drug therapy is a suitable option for patients seeking rapid relief.

**Trial registration:**

The study was registered at the Us Clinical Trial Registration Center (registration number: NCT06565845;date: 08/21/2024).

## Introduction

Granulomatous lobular mastitis (GLM), a rare inflammatory disease of the breast, was first described by Kessler and Wolloch in 1972 (1). The incidence of GLM has been rising annually in recent years, and is higher in the Mediterranean region and in Asian countries (2). It commonly affects one breast in women of childbearing age, and can occur in any quadrant of the breast (3). The primary clinical manifestation is the presence of a breast lump, which may or may not be accompanied by pain. Extra-mammary manifestations such as erythema nodosum and arthritis are rare (4). Ultrasound is the preferred imaging method for diagnosing GLM, and irregular hypoechoic masses and associated tubular hypoechoic areas are the most common findings (2).

Currently, the main treatment methods for GLM include surgery, drug therapy (corticosteroids, anti-tuberculosis drugs, immunosuppressants, and traditional Chinese medicine [TCM] formulations), the combination of surgery and drugs, and close observation. However, there is no standardized treatment protocol for GLM at present. Although surgical interventions can provide rapid relief, the postoperative recurrence rate reported in literature ranges from 5% to 50% (5–7). Although steroid therapies have improvement rates of 70%–80% (8, 9), their clinical application is limited because of potential side effects such as weight gain and Cushing syndrome (3). Previous studies revealed that methotrexate (MTX) therapy has an average recurrence-free remission rate of 79% (10). However, it is associated with severe side effects such as bone marrow suppression, interstitial pneumonia, and folate deficiency (11). Since Taylor et al. (12) first detected corynebacteria in GLM lesions in 2003, an increasing number of studies have found that GLM is associated with non-tuberculous mycobacteria and corynebacteria. Some recent small-sample studies have shown that triple anti-tuberculosis therapy is effective against GLM (13, 14), although the evidence is insufficient. Our study proposes that the triple anti-tuberculosis drug therapy can achieve the best therapeutic effect for GLM patients as it can avoid surgical trauma and minimize the impact on breast shape and daily life. GLM typically has a long disease course, and is difficult to treat and prone to recurrence. It also affects breast appearance and the overall well-being of patients. However, only a few studies have included the subjective experience of patients in the evaluation of treatment outcomes.

To date, no previous study has compared surgical treatment with triple anti-tuberculosis drug therapy in GLM patients, making this study the first to propose the combination of surgery and triple anti-tuberculosis drugs to treat GLM patients. Hence, we aimed to compare the efficacy and patient satisfaction of surgery, triple anti-tuberculosis drug therapy, and their combination in the same population of GLM patients, and provide new ideas for the clinical treatment.

## Methods

### Participants

GLM patients who received treatment at the Breast Surgery Department of the First Affiliated Hospital of Harbin Medical University from January 2019 to June 2023 were selected. The demographic data, clinical characteristics, treatment outcomes, patient satisfaction scores, and follow-up data of all patients were collected and retrospectively analyzed. The study was approved by the Ethics Committee of the First Affiliated Hospital of Harbin Medical University (No. 2024182) and conducted in accordance with the Declaration of Helsinki (revised 2013).

The inclusion criteria were as follows: histopathological confirmation of GLM, and normal liver and kidney function. The exclusion criteria were as follows: lactation or pregnancy, allergy to rifampin, isoniazid, or ethambutol, concurrent malignant breast tumors, severe underlying diseases, other conditions deemed unsuitable by the investigator, and refusal to participate in the study. All patients were diagnosed with GLM through core needle biopsy or excisional biopsy. Other possible granulomatous diseases, including Wegener’s granulomatosis, sarcoid-like granulomatous reactions, and foreign body granulomas, were excluded. Breast tuberculosis was excluded on the basis of medical history, lung CT, and histopathological examination.

### Treatment

The patients were divided into the following groups based on their treatment plans: group A - surgery; group B - triple anti-tuberculosis drug therapy; group C – combination of surgery and triple anti-tuberculosis drug therapy. Treatment plans were chosen according to the size of the GLM mass, ratio of the GLM mass to breast tissue, severity of disease, and the aesthetic concerns and personal preferences of the patients. The drawbacks of each option, such as breast deformity, scarring, side effects, and treatment duration, were also considered. Patients were thoroughly informed about the three treatment options, the treatment process, and potential adverse reactions, and the final plan was determined accordingly.

Surgical treatment involved complete excision of the inflammatory breast tissue, the damaged skin, and some surrounding normal tissue. If the diseased tissue invaded the major lactiferous ducts, the ducts behind the nipple were also excised. The surgical cavity was repeatedly irrigated with hydrogen peroxide, povidone–iodine, and saline, and a negative pressure drainage tube was placed before closing the incision. In case of inflammatory infiltration or multiple inflamed areas in the purulent skin, the incision was not sutured immediately. After the surgery, the incision was irrigated and dressed daily with hydrogen peroxide and povidone–iodine until the inflammation subsided, at which point the skin was sutured in a secondary procedure, and a negative pressure drainage tube was placed. The drainage volume and incision healing were monitored daily. The negative-pressure drainage tube was removed when the drainage volume was less than 1 mL for 3 consecutive days. The sutures were removed once the incision healed completely.

The anti-tuberculosis drug regimen consisted of oral rifampin (450 mg/day), isoniazid (300 mg/day), and ethambutol (15 mg/kg/day) for 6–9 months. Appropriate adjustments were made on the basis of clinical and ultrasound evaluations. The treatment was discontinued when clinical symptoms and ultrasound findings had been resolved for more than 1 month. Liver function was checked monthly during the treatment period. Small incision drainage or ultrasound-guided puncture drainage was performed for patients who developed abscesses. The treatment was discontinued in case of no improvement, worsening of symptoms, or severe adverse reactions, and other treatment options were considered according to the patient’s condition.

Patients in group C underwent surgery as described above, followed by anti-tuberculosis drug therapy for 1–3 months based on their preoperative condition. Patients who experienced mild pain and displayed a mass smaller than 3 cm without rupture or fistula during preoperative ultrasound received drug therapy for 1 month postoperatively. In addition, patients with severe pain and masses larger than 3 cm with or without rupture or fistula in the ultrasound findings received drug therapy for 3 months postoperatively. The dosage and administration of the drugs were the same as described above.

### Clinical evaluation

The clinical efficacy of the different treatment regimens was evaluated according to the following criteria: cure - complete disappearance of breast lesions in the post-treatment clinical and imaging examination; improvement - significant reduction in breast lesions compared with the pre-treatment status with significant relief in pain and swelling, and some patients presenting fistulas or sinus tracts; ineffective - lack of significant reduction in existing lesions, an increase in breast lesions compared with the pre-treatment status, or the appearance of new lesions, with persistent pain and swelling; recurrence - redness, swelling, pain, or the appearance of new lesions in the same breast after cure as confirmed by pathological examination. Adverse events (AEs) were evaluated according to the Common Adverse Event Evaluation Standard version 4.0, developed by the US Department of Health and Human Services.

### Satisfaction questionnaire

As GLM is a benign disease, treatment efficacy is evaluated in terms of quantitative indicators and the subjective feelings of patients. All cured patients were asked to complete the “patient satisfaction questionnaire” developed by Wang et al. (15)

### Statistical analysis

Statistical analyses were conducted using IBM SPSS 27.0. Categorical variables were presented as numbers and percentages, whereas continuous variables were summarized as mean ± standard deviation (or median and range when necessary). Pearson’s chi-squared test or Fisher’s exact test was used to compare categorical variables. The normality of the data distribution was assessed using the Kolmogorov–Smirnov test. The Kruskal–Wallis test was used to compare continuous variables when the data were non-normally distributed. All tests were considered statistically significant at p < 0.05.

## Results

### Demographic characteristics

**Figure 1:**
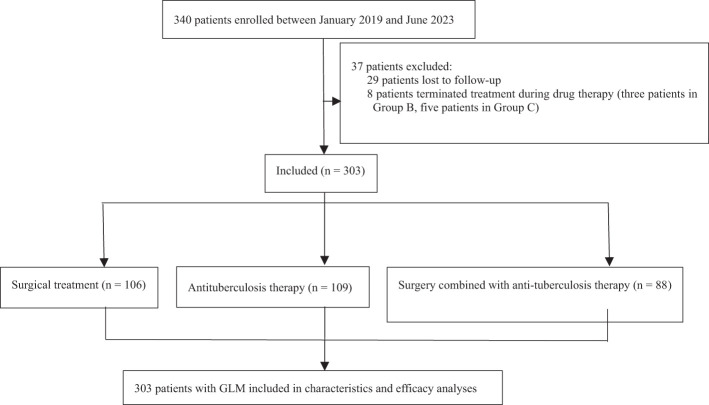
Flow diagram of patient enrollment. GLM, granulomatous lobular mastitis.

A total of 340 patients with GLM who received treatment from January 2019 to June 2023 at our center were initially selected. Twenty-nine patients were lost during follow-up, and eight patients discontinued treatment because of non-compliance (three in Group B, five in Group C). Finally, 303 patients were included in the study. The flowchart of patient enrollment is presented in **Figure 1**.

The median age of the patients was 32 years (range, 16–65 years). The demographic characteristics of the three treatment groups are presented in **Table 1**. There were no significant differences in the baseline characteristics among the three groups (p > 0.05). Analysis of the medical records revealed that 45 patients (14.9%) had a history of breast disease before the onset of GLM (14 cases of lactational mastitis, 25 cases of GLM, and 6 cases of benign breast tumor resection). Six patients (2%) had mental disorders, six patients (2%) had a history of autoimmune diseases (five cases of rheumatism and one case of ankylosing spondylitis), five patients (1.7%) had a history of oral contraceptive use, and three patients (1%) had a history of tuberculosis or exposure to tuberculosis.

**Table 1:** Demographic data and disease characteristics of patients with granulomatous lobular mastitis.

Demographic data and disease characteristics	All patients (N = 303)	Group A (N = 106)	Group B (N = 109)	Group C (N = 88)	P
Age (years), median (range)	32 (16–65)	32 (19–65)	33 (16–54)	32 (17–53)	0.473
BMI (kg/m^2^), mean (range)	25.39 (16.48–48.99)	25.72 (16.64–48.99)	24.58 (16.48–37.20)	25.99 (18.36–39.67)	0.099
Pregnancy history, n (%)	290 (95.7)	102 (96.2)	103 (94.5)	85 (96.6)	0.823
Number of births, mean (range)	1.15 (0–3)	1.16 (0–2)	1.09 (0–2)	1.20 (0–3)	0.291
Lactation, n (%)	257 (84.8)	86 (81.1)	96 (88.1)	75 (85.2)	0.370
Recent history of breast trauma, n (%)	171 (56.4)	58 (54.7)	67 (61.5)	46 (52.3)	0.393
Smoking, n (%)	14 (4.6)	5 (4.7)	4 (3.7)	5 (5.7)	0.745
Diameter of the target lesion (cm), mean ± SD	5.37 ± 2.63	4.87 ± 2.53	5.66 ± 2.55	5.61 ± 2.77	0.058

GLM usually manifests unilaterally, and the lesions can be located in any part of the breast. Furthermore, arthritis and lower-limb erythema nodosum are rare. All patients in our study cohort underwent ultrasonography (USG), but only 23 patients underwent mammography (MMG). The remaining patients did not undergo MMG because of pain or ulceration. Most patients exhibited heterogeneous hypoechoic areas and fluid-filled dark areas on USG, and mass lesions were the most common findings on MMG. The signs, symptoms, and USG findings are presented in **Table 2**.

**Table 2:** Signs, symptoms, ultrasonography findings, and initial therapeutic modality of patients with granulomatous lobular mastitis.

Variables	All patients (N = 303)	Group A (N = 106)	Group B (N = 109)	Group C (N = 88)
Side, n (%)				
Left	151 (49.8)	49 (46.2)	62 (52.9)	40 (45.5)
Right	144 (47.5)	54 (50.9)	45 (41.3)	45 (51.1)
Bilateral	8 (2.6)	3 (2.8)	2 (1.8)	3 (3.4)
Lesion location, n (%)				
Retroareolar	28 (9.2)	13 (12.3)	8 (7.3)	7 (8.0)
Upper outer quadrant	69 (22.8)	21 (19.8)	33 (30.3)	15 (17.0)
Lower outer quadrant	29 (9.6)	8 (7.5)	13 (11.9)	8 (9.1)
Upper inner quadrant	58 (19.1)	22 (20.8)	18 (16.5)	18 (20.5)
Lower inner quadrant	32 (10.6)	16 (15.1)	7 (6.4)	9 (10.2)
Multiple quadrants	87 (28.7)	26 (24.5)	30 (27.5)	31 (35.2)
Local manifestations, n (%)				
Mass	299 (98.7)	105 (99.1)	108 (99.1)	86 (97.7)
Pain	203 (67.0)	73 (68.9)	66 (60.6)	64 (72.7)
Abscess	108 (35.6)	39 (36.8)	46 (42.2)	23 (26.1)
Ulceration	96 (31.7)	31 (29.2)	41 (37.6)	24 (27.3)
Tenderness	252 (83.2)	94 (88.7)	87 (79.8)	71 (80.7)
Sinus formation	38 (12.5)	14 (13.2)	17 (15.6)	7 (8.0)
Inflammatory hyperemic skin	178 (58.7)	65 (61.3)	59 (54.1)	54 (61.4)
Breast discharge	33 (10.9)	12 (11.3)	8 (7.3)	13 (14.8)
Edema	182 (60.1)	64 (60.4)	63 (57.8)	55 (62.5)
Nipple retraction	130 (42.9)	43 (40.6)	47 (43.1)	40 (45.5)
Systemic symptoms, n (%)				
Arthralgia	27 (8.9)	6 (5.7)	12 (11.0)	9 (10.2)
Erythema nodosum	18 (5.9)	7 (6.6)	9 (8.3)	2 (2.3)
Fever	31 (10.2)	9 (8.5)	13 (11.9)	9 (10.2)
Ultrasonography findings, n (%)				
Heterogeneous hypoechoic areas	252 (83.2)	80 (75.5)	100 (91.7)	72 (81.8)
Collection	213 (70.3)	73 (68.9)	75 (68.8)	65 (73.9)
Axillary lymphadenopathy	120 (39.6)	38 (35.8)	39 (35.8)	43 (48.9)
Interstitial edema	112 (37.0)	34 (32.1)	50 (45.9)	28 (31.8)
Sinus formation	45 (14.9)	21 (19.8)	10 (9.2)	14 (15.9)
Duct ectasia	44 (14.5)	17 (16.0)	14 (12.8)	13 (14.8)
BI-RADS ratings of color ultrasound, n (%)				
BI-RADS 2	4 (1.3)	1 (0.9)	3 (2.8)	0
BI-RADS 3	202 (66.7)	67 (63.2)	81 (74.3)	54 (61.4)
BI-RADS 4a	81 (26.7)	28 (26.4)	22 (20.2)	31 (35.2)
BI-RADS 4b	14 (4.6)	9 (8.5)	3 (2.8)	2 (2.3)
BI-RADS 4c	2 (0.7)	1 (0.9)	0	1 (1.1)
Follow-up time (months), median (range)	35.43 (13.27–67.90)	40.65 (14.53–67.90)	27.80 (13.26–65.07)	39.68 (13.83–66.53)

TCM, Traditional Chinese medicine.

### Efficacy evaluation

The cutoff date for our retrospective analysis was July 31, 2024, and the median follow-up duration was 35.43 months (range, 13.27–67.90 months). As shown in **Figure 1**, the patients were grouped and analyzed according to the intention-to-treat principle. Forty-nine patients in group B underwent ultrasound-guided puncture drainage or small incision drainage during the abscess phase. The symptoms of arthritis and lower-limb erythema nodosum in 36 patients resolved as the disease subsided. The median treatment durations in groups A, B, and C were 14 days (range, 7–90 days), 240 days (range, 30–450 days), and 90 days (range, 30–90 days) respectively, and the differences among the groups were significant (P < 0.001). According to the efficacy evaluation criteria, cure rates did not significantly differ among the groups (P > 0.05) and exceeded 90% in all groups. During the follow-up period, 24 patients in group A (22.6%), 4 patients in group B (3.7%), and 8 patients in group C (9.1%) experienced recurrence. The recurrence rates were significant lower in groups B and C compared to that in group A (P < 0.001 and P = 0.019 respectively), whereas the difference between groups B and C was not significant (P = 0.212). The results of efficacy evaluation are summarized in **Table 3**.

**Table 3:** Outcomes of therapeutic modalities.

Variables	Group A (N = 106)	Group B (N = 109)	Group C (N = 88)	P
Complete response, n (%)	102 (96.2)	100 (91.7)	85 (96.6)	0.220
Partial response, n (%)	2 (1.9)	4 (3.7)	3 (3.4)	0.762
No response, n (%)	2 (1.9)	5 (4.6)	0	0.138
Relapse, n (%)	24 (22.6) △*	4 (3.7)	8 (9.1)	<0.001
Treatment duration (days), median (range)	14 (7–90) △*	240 (30–450)*	90 (30–90)	<0.001

△ Compared with group B, P < 0.05; * compared with group C, P < 0.05.

### Safety analysis

No Serious Adverse events (SAEs) were observed among the 197 patients who received anti-tuberculosis drugs. However, 26 patients (13.20%) experienced treatment-related AEs, including liver dysfunction (n = 12), gastrointestinal reactions (n = 8), rash and itching (n = 2), fatigue (n = 1), a mild decrease in platelet counts (n = 1), mild blurred vision (n = 1), and menstrual disorder (n = 1). The adverse reactions were well tolerated and disappeared within 1 month after stopping treatment. Hepatoprotective tablets were prescribed for patients with liver dysfunction, which led to improvements in symptoms. Among the 194 patients who underwent surgery, 20 (10.3%) experienced postoperative complications, including delayed wound healing (n = 10), fistula formation (n = 4), subcutaneous fluid accumulation (n = 4), and wound dehiscence (n = 2). All complications were well managed and resolved within 3 months.

### Satisfaction questionnaire analysis

The overall satisfaction scores of the cured patients significantly differed among the three groups (P < 0.001; **Table 4**). The total satisfaction score was higher in group B (46.44 ± 3.21) than in groups A (41.84 ± 4.54) and C (41.81 ± 3.91, both P < 0.001), whereas the scores were similar in groups A and C (P > 0.05). Overall, patients in group B reported the highest scores for total satisfaction, breast appearance, treatment cost, quality of life, and treatment efficacy.

**Table 4:** Satisfaction scores for the three treatment modalities.

Satisfaction scores	Group A (N = 102)	Group B (N = 100)	Group C (N = 85)	P
Total	41.84 ± 4.54	46.44 ± 3.21△*	41.81 ± 3.91	<0.001
Breast shape	6.78 ± 1.60	9.50 ± 0.87△*	6.54 ± 1.49	<0.001
Treatment time	9.18 ± 1.27	8.50 ± 1.43△	8.80 ± 1.42	<0.001
Economic costs	9.22 ± 1.30	9.92 ± 0.39△*	9.34 ± 1.25	<0.001
Treatment effect	9.00 ± 1.84*	9.80 ± 1.01△	9.62 ± 1.18	<0.001
Life influence	7.67 ± 1.38	8.72 ± 1.79△*	7.51 ± 1.38	<0.001

△ Compared with group A, P < 0.05; * compared with group C, P < 0.05.

## Discussion

Because of its rarity, the current literature on GLM is limited to case reports or small-sample retrospective studies, which limits the validity of the results. Our study is the first single-center, large-sample analysis of different treatment methods in a cohort of GLM patients.

GLM typically occurs 2–6 years after childbirth (16), and rarely affects pregnant or lactating women (2). Although its exact etiology is unclear, autoimmunity, infection, and hormonal imbalance have been implicated as potential causes. In addition, obesity, pregnancy, breastfeeding, nipple retraction, a history of blunt breast trauma, antipsychotic medications, smoking, and oral contraceptives are possible risk factors (3, 17, 18). In this study, 95.7% of the patients had a history of pregnancy, 84.8% had a history of breastfeeding, 56.4% had a history of blunt breast trauma before onset, and 42.9% had nipple retraction. The average BMI of the cohort was ≥23.9 kg/m2. These results are consistent with previous findings and suggest that GLM can be triggered by hormonal imbalances and other chemical stimuli that increase ductal permeability, or by blunt trauma that causes ductal epithelial damage. These changes result in the leakage of accumulated milk or ductal secretions into the lobular connective tissue, leading to local stromal inflammation, immune cell infiltration, and eventually the formation of local granulomas (3, 19).

Surgery can rapidly alleviate the symptoms of GLM and therefore has long been the preferred treatment. GLM often presents as a rapidly developing breast lump with or without pain, which can progress to abscess formation, fistulas, and skin ulcerations (5, 15, 20). Overall, 98.7% of the patients in our cohort presented with breast lumps with or without pain, whereas extra-mammary manifestations such as arthritis and erythema nodosum in the lower limbs were observed in only 8.9% and 5.9% of patients respectively. This is consistent with previous reports (4). Although extra-mammary symptoms such as arthritis and erythema nodosum are suggestive of an autoimmune disorder, GLM predominantly affects one breast (3). Consistent with this, only 2.6% of the cases in our study were bilateral. The clinical presentation of GLM is similar to that of inflammatory breast cancer, and its imaging features are difficult to distinguish from those of breast tumors (21, 22). Therefore, the gold standard for diagnosing GLM is biopsy of the lesion. Among the 303 patients included in our study, 32.0% had a Breast Imaging Reporting and Data System (BI-RADS) ultrasound score greater than 3, further highlighting the necessity of biopsy. Due to local signs of inflammation such as redness, swelling, heat, and pain, many GLM patients initially receive empirical antibiotic treatment. However, traditional antibiotics are generally ineffective (14).

However, the postoperative recurrence rate ranges between 5%–50% (5–7). Additionally, the breast shape can be severely compromised in patients with extensive lesions or an acute inflammatory response. Therefore, the relative contraindications for surgery are lesions involving more than two-thirds of the breast and severe skin involvement (2). In this study, patients who received triple anti-tuberculosis drug therapy had the highest satisfaction scores regarding breast appearance (P < 0.001), indicating that drugs have less impact on breast appearance than surgery. To improve treatment outcomes, multiple treatment methods are often used concurrently or sequentially. Previous studies have shown that postoperative sequential corticosteroids can reduce recurrence rates in patients with refractory or severe GLM (23). In our study, 88 patients received postoperative sequential triple anti-tuberculosis drug therapy, which decreased the recurrence rate to 9.1%, compared with 22.6% in patients who only underwent surgery. This offers a new approach for addressing the high postoperative recurrence rate associated with surgery alone. Future studies might consider extending the duration of postoperative oral triple anti-tuberculosis drug therapy to determine whether it further reduces the recurrence rate. However, research into the underlying mechanisms by which combination therapy reduces recurrence rates was lacking in our study. The possible reason that combination therapy can reduce recurrence is that triple anti-tuberculosis drugs can inhibit lipophilic corynebacterium, reducing the probability of short-term recurrence after surgery. The specific mechanism is worth exploring in future studies.

The outcomes of triple anti-tuberculosis drug therapy at our center were consistent with recent studies that have all demonstrated excellent efficacy. For instance, Liu et al. (13) reported a complete remission rate of 94.7% in patients with GLM (18/19) after triple anti-tuberculosis therapy. Likewise, in a study conducted by Zhou et al. (14), anti-tuberculosis drugs achieved a cure rate of 78.51% in GLM patients, with a recurrence rate of only 9.76%. In our study, the cure rate of triple antituberculosis drug treatment in GLM patients was 91.7%, and the recurrence rate was 3.7%. In addition, no SAEs were noted in our study, and treatment-related AEs (13.2%) disappeared shortly after treatment discontinuation. On the other hand, long-term glucocorticoid use is associated with serious side effects such as Cushing syndrome, osteoporosis, glucose intolerance, gastrointestinal reactions, and secondary infections (2, 3). Altogether, given the side effects and contraindications associated with systemic corticosteroid use, anti-tuberculosis drugs can be considered effective alternatives. Furthermore, triple anti-tuberculosis drug therapy is more cost-effective than either surgery or glucocorticoid therapy. Patients avoid paying for surgery and hospitalization, with little impact on daily work. All patients are able to afford medication during treatment, reducing the burden on the healthcare system and the patients themselves. Previous literature has suggested that patients with GLM who do not have a response to steroids can be treated with MTX alone or in combination with steroids (24). Therefore, in future studies, MTX alone or in combination with triple anti-tuberculosis drugs can be tried for GLM patients with poor response to triple anti-tuberculosis drugs.

The relationship between GLM and corynebacteria, particularly *Corynebacterium kroppenstedtii*, has become increasingly evident in recent years, suggesting that corynebacteria play a significant role in the development of GLM. Since Taylor et al. (12) first detected *C. kroppenstedtii* in GLM lesions, several groups have isolated and cultivated corynebacteria. For example, Paviour et al. (25) detected *Corynebacterium* in the breast lesions of 12 out of 24 patients through pathological assessment, and diagnosed nine of those cases as GLM. Furthermore, Omar et al. (26) confirmed that rifampin, a lipophilic antibiotic, is effective against corynebacteria, which might explain the effectiveness of triple anti-tuberculosis drug therapy in GLM patients. In a previous small-sample study (26), 30 patients with GLM were cured after 12 months of treatment with rifampin. Therefore, it is worth investigating whether reducing the drug dosage or switching to one or two anti-tuberculosis drugs can improve outcomes in patients with GLM.

There are no reports of GLM progressing to a malignant stage (18). Nevertheless, patients commonly experience significant physical and psychological distress during and after treatment because of breast disfigurement, along with economic, familial, and health-related pressures (27). Therefore, the subjective perceptions of patients are as important as quantitative indicators when evaluating treatment methods for GLM. Patient satisfaction was highest for triple anti-tuberculosis drug therapy, which is most likely attributable to its negligible effect on overall breast appearance. Furthermore, the drug regimen is cost-effective, does not require hospitalization, and has a minimal impact on patients’ work and lives. However, long-term drug usage can lead to patient fatigue and reduced adherence. The lower satisfaction in the surgery group might be attributable to breast deformity, high postoperative recurrence rates, and a prolonged inability to work and perform daily activities after surgery, which lowers patients’ quality of life. With the development of new treatments, The researchers developed an innovative multifunctional drug delivery system for magnetic resonance (MR) imaging-mediated anti-tumor therapy (28). This provides new ideas for future medical treatment.

This study had several limitations, including its retrospective nature, the lack of a standardized evaluation system, and the inability to obtain results for patients lost to follow-up. The choice of treatment modality takes into account the individual preferences of patients, leading to potential selection bias that may interfere with the choice of the correct treatment modality. Recall bias may exist in the survey of patient satisfaction, which may lead to inaccurate recall of satisfaction scores by patients. In addition, no tests were performed to identify *Corynebacterium* in any of the patients. Given the lack of a unified standard for evaluating treatment efficacy in GLM patients, we determined the outcomes based on our own experience. Multicenter, prospective, randomized controlled trials are needed to help develop standardized treatment protocols for GLM and explore its etiology.

## Conclusions

Triple anti-tuberculosis drug therapy is an effective treatment for GLM with good clinical efficacy, low recurrence rate and high patient satisfaction, which has a promising clinical application prospect. For rapid relief, combining surgery with triple anti-tuberculosis drug therapy is effective, and combination therapy can solve the problem of high recurrence rate after surgery alone.

## Data Availability

The raw data supporting the conclusions of this article will be made available by the authors, without undue reservation.
